# μ_3_-Dodeca­tungsto(V,VI)aluminato-κ^3^ *O*:*O*′:*O*′′-tris­[aqua­bis­(ethyl­ene­diamine-κ^2^ *N*,*N*′)copper(II)]

**DOI:** 10.1107/S1600536811048288

**Published:** 2011-11-19

**Authors:** Yu-Kun Lu, Yuan-Yuan Qu, Ming-Ming Tian, Cheng-Lin Diao, Yun-Qi Liu

**Affiliations:** a State Key Laboratory of Heavy Oil Processing, College of Science, China University of Petroleum (East China), Qingdao Shandong 266555, People’s Republic of China; b State Key Laboratory of Heavy Oil Processing, College of Chemical Engineering, China University of Petroleum (East China), Qingdao Shandong 266555, People’s Republic of China

## Abstract

The title compound, [AlCu_3_W_12_O_40_(C_2_H_8_N_2_)_6_(H_2_O)_3_], was prepared under hydro­thermal conditions. The Cu^2+^ ion displays an elongated octa­hedral geometry defined by one bridging O atom from the polyoxidoanion and a coordinated water mol­ecule in axial positions and four N atoms of the two chelating ethyl­enediamine (en) ligands in equatorial positions. The one-electron reduced [AlW_12_O_40_]^6−^ anion coordinates three [Cu(en)(H_2_O)]^2+^ fragments, generating a neutral tri-supported Keggin-type polyoxidometalate (POM). This tri-supported POM is located in a special position of 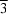 symmetry and therefore O atoms from the central AlO_4_ tetra­hedron are disordered over two sets of sites. Disorder is also observed for three other bridging O atoms of the POM. In the crystal, mol­ecules are connected *via* N—H⋯O and O—H⋯O hydrogen bonds, forming a three-dimensional framework.

## Related literature

For the isotypic V^IV^ and Si^IV^ structures, see: Lu, Cui, Chen *et al.* (2009 ▶). For general background to polyoxidometalates, see: Pope & Müller (1991 ▶); Hill (1998 ▶); López *et al.* (2001 ▶). For modified Keggin-type structures with transition metal complexes, see: Xu *et al.* (2000 ▶); Yuan, Li *et al.* (2003 ▶). For the structure and chemistry of one-electron reduced heteropolytungstate, see: Lan *et al.* (2008 ▶); Meng *et al.* (2008 ▶). For other dodeca­tungstoaluminates, see: Wang *et al.* (2006 ▶); Yuan, Qin *et al.* (2009 ▶). For polyoxidometalates prepared with strongly reducing agents, see: Lu, Cui, Liu *et al.* (2009 ▶); Lu, Xu & Yu (2010 ▶); Lu, Xu, Cui *et al.* (2010 ▶).
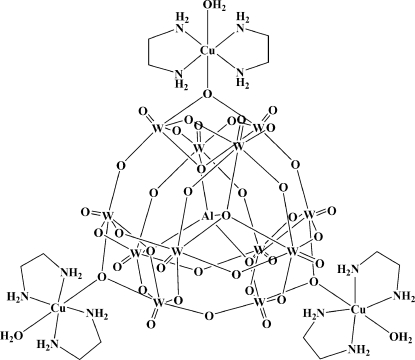


## Experimental

### 

#### Crystal data

[AlCu_3_W_12_O_40_(C_2_H_8_N_2_)_6_(H_2_O)_3_]
*M* *_r_* = 3478.47
Trigonal, 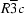

*a* = 17.9719 (14) Å
*c* = 29.335 (5) Å
*V* = 8206 (2) Å^3^
*Z* = 6
Mo *K*α radiation
μ = 26.38 mm^−1^
*T* = 296 K
0.11 × 0.11 × 0.10 mm

#### Data collection

Rigaku R-AXIS RAPID diffractometer
Absorption correction: multi-scan (*ABSCOR*; Higashi, 1995 ▶) *T*_min_ = 0.159, *T*_max_ = 0.178
22760 measured reflections
2220 independent reflections
1864 reflections with *I* > 2σ(*I*)
*R*_int_ = 0.071

#### Refinement

*R*[*F* ^2^ > 2σ(*F* ^2^)] = 0.035
*wR*(*F* ^2^) = 0.071
*S* = 1.10
2220 reflections
157 parameters
H-atom parameters constrained
Δρ_max_ = 1.85 e Å^−3^
Δρ_min_ = −3.56 e Å^−3^

### 

Data collection: *RAPID-AUTO* (Rigaku, 1998 ▶); cell refinement: *RAPID-AUTO*; data reduction: *RAPID-AUTO*; program(s) used to solve structure: *SHELXS97* (Sheldrick, 2008 ▶); program(s) used to refine structure: *SHELXL97* (Sheldrick, 2008 ▶); molecular graphics: *DIAMOND* (Brandenburg, 1999 ▶); software used to prepare material for publication: *SHELXL97*.

## Supplementary Material

Crystal structure: contains datablock(s) I, global. DOI: 10.1107/S1600536811048288/gk2416sup1.cif

Structure factors: contains datablock(s) I. DOI: 10.1107/S1600536811048288/gk2416Isup2.hkl

Additional supplementary materials:  crystallographic information; 3D view; checkCIF report

## Figures and Tables

**Table 1:** Hydrogen-bond geometry (Å, °)

*D*—H⋯*A*	*D*—H	H⋯*A*	*D*⋯*A*	*D*—H⋯*A*
O1*W*—H1*W*⋯O2^i^	0.85	2.25	2.856 (9)	128
N1—H1*B*⋯O5^i^	0.90	2.26	3.138 (17)	163
N1—H1*B*⋯O5′^i^	0.90	2.30	3.185 (17)	170
N2—H2*A*⋯O7^ii^	0.90	2.35	3.101 (17)	141
N2—H2*B*⋯O1^iii^	0.90	2.11	2.956 (12)	157

Symmetry codes: (i) 
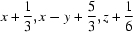
; (ii) 
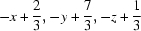
; (iii) 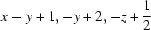.

**Fig. 1.**
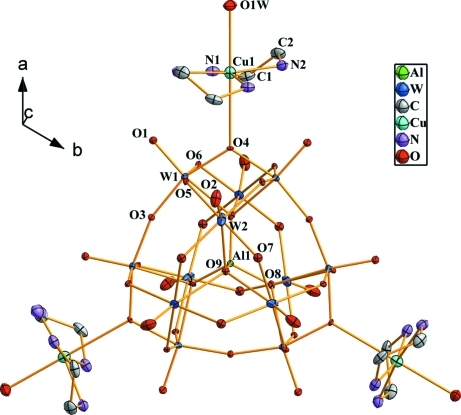
The molecular structure of the title compound with displacement ellipsoids drawn at the 30% probability level and labels shown for the asymmetric unit; the remaining part of the molecule was generated by the symmetry operations: -y, x-y, z; -x+y,-x, z; x-y, -y, 0.5-z; -x, -x+y, 0.5-z; y, x, 0.5-z. Only one position of the {AlO4} unit and one position of the disordered O5, O6 and O7 atoms is shown. H atoms have been omitted for clarity.

**Fig. 2.**
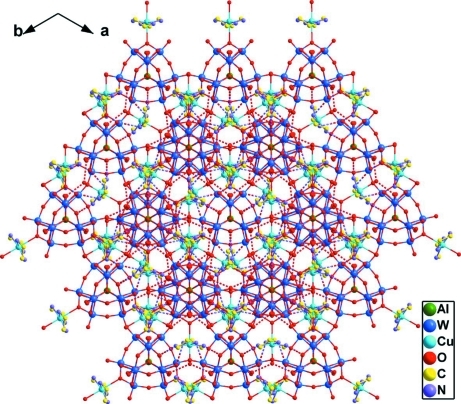
The crystal packing of the title compound viewed along the [001] direction with N—H···O and O—H···O hydrogen bonds displayed as dashed lines.

## supplementary crystallographic information

### Comment

There has been extensive interest in polyoxometalates (POMs), owing to their fascinating properties and great potential applications in many fields including catalysis, material science, medicine, and magnetochemistry, and their unusual structural diversities (Pope & Müller, 1991; Hill, 1998; López *et al.*, 2001). Especially, the modified POMs, which are the decoration of polyoxoanions with various transition metal ions, organic ligands, and/or their complex moieties, can be regarded as an ideal atomic level structural model for the determination of the mechanisms of oxide-supported catalysts (Xu *et al.*, 2000; Yuan, Li *et al.*, 2003). Therefore, we focussed our research on the preparation of modified POMs with strong reducing reagents (Lu, Cui, Liu *et al.*, 2009; Lu, Xu, Cui *et al.*, 2010, Lu, Xu, Yu *et al.*, 2010).

As shown in Fig. 1, the title compound shows a neutral tri-supported classical psedo-Keggin type structure where three [Cu(en)_2_(H_2_O)]^2+^ fragments are decorating the one-electron reduced heteropolyanion [AlW^VI^_11_W^V^O40]^6-^, which is isotypic with its V^IV^ and Si^IV^ analogue (Lu, Cui, Chen *et al.*, 2009). The tri-supported POM is located in a special position of 3 symmetry and therefore oxygen atoms from the central AlO_4_ tetrahedron are disordered over two sites. The pseudo-Keggin unit [AlW_12_O_40_]^6-^ may be viewed as a shell of {W_12_O_36_} encapsulating a disordered {AlO_4_} moiety, present at its center and responsible for the local tetrahedral geometry (Wang *et al.*, 2006; Yuan, Qin *et al.*, 2009). The central Al atom is surrounded by a cube of eight oxygen (six O8 and two O9) atoms with each of them having half-occupancy due to the inversion symmetry at Al1, and the oxygens of the {AlO_4_} group are covalently bonded to three different tungsten centers of the shell. All W atoms possess similar distorted octahedral geometry WO_6_ defined by one terminal oxygen atom, four doubly bridging oxo-groups and one central oxygen atom. Three doubly-bridging oxo-groups are disordered over two sets of sites each (O5, O5', O6, O6', O7 and O7') with the occupancy factor assigned as 0.5. A l—O8 and Al—O9 bond lengths are 1.714 (11) and 1.81 (2), respectively, with mean bond distance 1.74 Å, in good agreement with the literature (López *et al.*, 2001). The three classes of W—O average distances (being 1.688, 1.939 and 2.297 Å, respectively) are comparable to the corresponding distances in the similar structures (Wang *et al.*, 2006; Yuan, Qin *et al.*, 2009). The heteropolyanion [AlW^VI^_11_W^V^O_40_]^6-^ is a one-electron-reduced derivative of [AlW_12_O_40_]^6-^, similar to other reported representatives (Lan *et al.*, 2008; Meng *et al.*, 2008). We consider that oxalic acid acts as reducing agent reducing W^VI^ to W^V^ in the reactions.

The most unusual structural feature of the title compound is that each of three surface bridging oxygen atoms (O4) of the polyoxoanion is coordinated to one [Cu(en)_2_(H_2_O)]^2+^ fragment. The Cu1 center possesses an elongated octahedral geometry defined by the bridging oxygen atom (Cu—O4, 2.718 (9) Å) from the polyoxoanion, a coordination water molecule [Cu—O1W, 2.411 (11) Å] *trans* to O4 atom and four N atoms from two chelating en ligands with equal Cu—N bond lengths 2.002 (9) Å. The bond lengths and angles at Cu1 are consistent with the Jahn–Teller active d^9^ electronic configuration of divalent copper. The tri-supported POMs are extended into three-dimensional supramolecular network *via* a combination of intermolecular N—H···O and O—H···O hydrogen bonding (Fig. 2).

### Experimental

A mixture of Na_2_WO_4_.2H_2_O (0.658 g, 2 mmol), CuSO_4_.5H_2_O (0.25 g, 1 mmol), H_2_C_2_O_4_.2H_2_O (0.189 g, 1.5 mmol), NaAlO_2_ (0.10 g, 1.25 mmol) and H_2_O (15 mL) was mixed and stirred for 30 min, and the pH was adjusted to 7 with en. The resulting suspension was transferred to a Teflon-lined autoclave (25 ml) and kept at 180°C for 3 days. After slow cooling to room temperature for 2 days, blue prism crystals were obtained by filtering, washing with distilled water, and drying in desiccators at ambient temperature. The yields were *ca* 42% based on W. Elemental analysis C_12_H_54_Cu_3_N_12_O_43_AlW_12_(3478.47): Calcd. (%): C, 4.14; H, 1.56; N, 4.83. Found: C, 4.21; H, 1.57; N, 4.94.

### Refinement

H atoms bonded to C and N atoms were positioned geometrically and refined as riding atoms, with C—H = 0.97, N—H = 0.90 Å and *U*_iso_(H) = 1.2*U*_eq_(C, N). H atoms attached to the water molecule were located in a difference Fourier map and refined as riding, with O—H = 0.85 Å and *U*_iso_(H) = 1.5*U*_eq_(O). In the final difference Fourier map, the highest peak and the deepest hole are 0.37 Å and 0.93 Å from atom W2, respectively.

### Crystal data

**Table:**

[AlCu_3_W_12_O_40_(C_2_H_8_N_2_)_6_(H_2_O)_3_]	*D*_x_ = 4.224 Mg m^−^^3^
*M**_r_* = 3478.47	Mo *K*α radiation, λ = 0.71073 Å
Trigonal, *R*3*c*	Cell parameters from 4488 reflections
Hall symbol: -R 3 2"c	θ = 2.3–27.9°
*a* = 17.9719 (14) Å	µ = 26.38 mm^−^^1^
*c* = 29.335 (5) Å	*T* = 296 K
*V* = 8206 (2) Å^3^	Prism, blue
*Z* = 6	0.11 × 0.11 × 0.10 mm
*F*(000) = 9252	

### Data collection

**Table:**

Rigaku R-AXIS RAPID diffractometer	2220 independent reflections
Radiation source: fine-focus sealed tube	1864 reflections with *I* > 2σ(*I*)
graphite	*R*_int_ = 0.071
Detector resolution: 10 pixels mm^-1^	θ_max_ = 28.0°, θ_min_ = 2.3°
ω scans	*h* = −23→23
Absorption correction: multi-scan (*ABSCOR*; Higashi, 1995)	*k* = −23→23
*T*_min_ = 0.159, *T*_max_ = 0.178	*l* = −38→38
22760 measured reflections	

### Refinement

**Table:**

Refinement on *F*^2^	Secondary atom site location: difference Fourier map
Least-squares matrix: full	Hydrogen site location: inferred from neighbouring sites
*R*[*F*^2^ > 2σ(*F*^2^)] = 0.035	H-atom parameters constrained
*wR*(*F*^2^) = 0.071	*w* = 1/[σ^2^(*F*_o_^2^) + (0.0089*P*)^2^ + 625.9202*P*] where *P* = (*F*_o_^2^ + 2*F*_c_^2^)/3
*S* = 1.10	(Δ/σ)_max_ = 0.001
2220 reflections	Δρ_max_ = 1.85 e Å^−^^3^
157 parameters	Δρ_min_ = −3.56 e Å^−^^3^
0 restraints	Extinction correction: *SHELXL97* (Sheldrick, 2008), Fc^*^=kFc[1+0.001xFc^2^λ^3^/sin(2θ)]^-1/4^
Primary atom site location: structure-invariant direct methods	Extinction coefficient: 0.000015 (2)

### Special details

**Table:**

Geometry. All e.s.d.'s (except the e.s.d. in the dihedral angle between two l.s. planes) are estimated using the full covariance matrix. The cell e.s.d.'s are taken into account individually in the estimation of e.s.d.'s in distances, angles and torsion angles; correlations between e.s.d.'s in cell parameters are only used when they are defined by crystal symmetry. An approximate (isotropic) treatment of cell e.s.d.'s is used for estimating e.s.d.'s involving l.s. planes.
Refinement. Refinement of *F*^2^ against ALL reflections. The weighted *R*-factor *wR* and goodness of fit *S* are based on *F*^2^, conventional *R*-factors *R* are based on *F*, with *F* set to zero for negative *F*^2^. The threshold expression of *F*^2^ > σ(*F*^2^) is used only for calculating *R*-factors(gt) *etc*. and is not relevant to the choice of reflections for refinement. *R*-factors based on *F*^2^ are statistically about twice as large as those based on *F*, and *R*– factors based on ALL data will be even larger.

### Fractional atomic coordinates and isotropic or equivalent isotropic displacement parameters (Å^2^)

**Table:**

	*x*	*y*	*z*	*U*_iso_*/*U*_eq_	Occ. (<1)
Al1	0.0000	1.0000	0.2500	0.0086 (12)	
W1	0.12019 (2)	0.89540 (2)	0.251326 (13)	0.01398 (11)	
W2	0.11116 (3)	0.99748 (3)	0.151770 (19)	0.03112 (15)	
Cu1	0.38149 (11)	1.0000	0.2500	0.0250 (4)	
O1	0.1553 (4)	0.8230 (4)	0.2512 (3)	0.035 (2)	
O2	0.1623 (6)	0.9928 (5)	0.1054 (3)	0.035 (2)	
O3	0.0000	0.8191 (5)	0.2500	0.032 (3)	
O4	0.2303 (5)	1.0000	0.2500	0.021 (2)	
O5	0.1345 (9)	0.9100 (9)	0.1856 (6)	0.017 (3)	0.50
O5'	0.0992 (10)	0.9148 (9)	0.1888 (5)	0.014 (3)	0.50
O6	0.1377 (9)	0.9148 (8)	0.3158 (5)	0.016 (3)	0.50
O6'	0.0980 (9)	0.9157 (9)	0.3118 (5)	0.013 (3)	0.50
O7	0.0887 (8)	1.0866 (8)	0.1188 (5)	0.016 (3)*	0.50
O7'	−0.0036 (9)	0.9157 (8)	0.1502 (5)	0.018 (3)	0.50
O8	0.0000 (8)	1.0914 (7)	0.2333 (4)	0.010 (2)	0.50
O9	0.0000	1.0000	0.1883 (7)	0.011 (4)	0.50
O1W	0.5157 (6)	1.0000	0.2500	0.039 (3)	
H1W	0.5318	0.9756	0.2693	0.059*	
C1	0.3609 (8)	1.0205 (8)	0.3444 (4)	0.036 (3)	
H1C	0.3626	1.0033	0.3755	0.043*	
H1D	0.3169	1.0365	0.3420	0.043*	
C2	0.4485 (8)	1.0956 (8)	0.3305 (4)	0.037 (3)	
H2C	0.4615	1.1460	0.3484	0.044*	
H2D	0.4930	1.0811	0.3359	0.044*	
N1	0.3427 (6)	0.9487 (6)	0.3121 (3)	0.028 (2)	
H1A	0.2861	0.9102	0.3118	0.033*	
H1B	0.3712	0.9218	0.3208	0.033*	
N2	0.4457 (6)	1.1133 (6)	0.2820 (3)	0.031 (2)	
H2A	0.4994	1.1439	0.2707	0.037*	
H2B	0.4189	1.1438	0.2780	0.037*	

### Atomic displacement parameters (Å^2^)

**Table:**

	*U*^11^	*U*^22^	*U*^33^	*U*^12^	*U*^13^	*U*^23^
Al1	0.0072 (17)	0.0072 (17)	0.011 (3)	0.0036 (8)	0.000	0.000
W1	0.01112 (18)	0.00943 (17)	0.0236 (2)	0.00683 (14)	−0.00119 (14)	−0.00112 (14)
W2	0.0239 (2)	0.0154 (2)	0.0504 (3)	0.00709 (17)	0.0232 (2)	−0.00150 (19)
Cu1	0.0300 (7)	0.0256 (9)	0.0178 (9)	0.0128 (5)	0.0001 (4)	0.0001 (7)
O1	0.012 (3)	0.013 (3)	0.080 (6)	0.007 (3)	−0.003 (4)	−0.001 (4)
O2	0.056 (5)	0.023 (4)	0.022 (4)	0.017 (4)	0.013 (4)	0.003 (3)
O3	0.008 (4)	0.009 (3)	0.078 (9)	0.004 (2)	0.002 (5)	0.001 (3)
O4	0.005 (3)	0.010 (4)	0.049 (7)	0.005 (2)	−0.001 (2)	−0.003 (4)
O5	0.010 (7)	0.007 (6)	0.034 (9)	0.004 (6)	0.006 (7)	−0.001 (6)
O5'	0.015 (8)	0.015 (7)	0.009 (7)	0.005 (6)	−0.006 (6)	−0.004 (5)
O6	0.011 (7)	0.007 (6)	0.029 (8)	0.005 (6)	0.003 (6)	0.001 (5)
O6'	0.013 (7)	0.016 (7)	0.014 (7)	0.009 (6)	0.000 (6)	0.003 (5)
O7'	0.023 (7)	0.009 (6)	0.025 (8)	0.009 (6)	−0.004 (6)	0.002 (6)
O8	0.011 (5)	0.015 (6)	0.009 (5)	0.009 (5)	0.001 (6)	−0.004 (5)
O9	0.011 (6)	0.011 (6)	0.012 (10)	0.005 (3)	0.000	0.000
O1W	0.026 (4)	0.038 (7)	0.058 (8)	0.019 (3)	0.009 (3)	0.017 (6)
C1	0.044 (7)	0.062 (8)	0.022 (6)	0.042 (7)	0.001 (5)	0.001 (6)
C2	0.039 (7)	0.049 (8)	0.028 (6)	0.026 (6)	−0.014 (5)	−0.012 (6)
N1	0.025 (5)	0.040 (5)	0.024 (5)	0.020 (4)	0.002 (4)	0.002 (4)
N2	0.030 (5)	0.032 (5)	0.035 (6)	0.020 (4)	−0.008 (4)	−0.004 (4)

### Geometric parameters (Å, °)

**Table:**

Al1—O8^i^	1.714 (11)	W2—O7^iii^	2.079 (13)
Al1—O9	1.81 (2)	W2—O9	2.288 (10)
W1—O1	1.707 (7)	W2—O8^iii^	2.421 (11)
W1—O3	1.894 (6)	Cu1—N2	2.002 (9)
W1—O6'	1.894 (6)	Cu1—N1	2.002 (9)
W1—O6	1.921 (16)	Cu1—O1W	2.411 (11)
W1—O4	1.931 (4)	Cu1—O4	2.718 (9)
W1—O5'	1.939 (14)	O1W—H1W	0.8499
W1—O5	1.946 (17)	C1—N1	1.498 (15)
W1—O8^ii^	2.232 (11)	C1—C2	1.530 (17)
W1—O8^iii^	2.248 (12)	C1—H1C	0.9700
W2—O2	1.668 (8)	C1—H1D	0.9700
W2—O5'	1.765 (14)	C2—N2	1.465 (15)
W2—O7'^iv^	1.787 (13)	C2—H2C	0.9700
W2—O6'^v^	1.793 (14)	C2—H2D	0.9700
W2—O7'	1.840 (13)	N1—H1A	0.9000
W2—O6^v^	2.063 (14)	N1—H1B	0.9000
W2—O5	2.072 (15)	N2—H2A	0.9000
W2—O7	2.076 (13)	N2—H2B	0.9000
			
O8^ii^—Al1—O8^i^	112.2 (3)	O6'^v^—W2—O8^iii^	56.2 (5)
O8^ii^—Al1—O8^iv^	122.8 (8)	O7'—W2—O8^iii^	85.6 (5)
O8^ii^—Al1—O9^ii^	73.4 (4)	O6^v^—W2—O8^iii^	68.1 (5)
O9^ii^—Al1—O9	180.000 (2)	O5—W2—O8^iii^	68.2 (5)
O1—W1—O3	99.8 (4)	O7—W2—O8^iii^	111.0 (5)
O1—W1—O6'	110.1 (5)	O7^iii^—W2—O8^iii^	112.0 (5)
O3—W1—O6'	83.4 (5)	O9—W2—O8^iii^	53.0 (5)
O1—W1—O6	93.0 (5)	N2—Cu1—N2^v^	172.2 (5)
O3—W1—O6	100.1 (4)	N2—Cu1—N1	86.2 (4)
O6'—W1—O6	22.1 (4)	N2^v^—Cu1—N1	94.7 (4)
O1—W1—O4	98.8 (3)	N2—Cu1—N1^v^	94.7 (4)
O3—W1—O4	161.2 (3)	N2^v^—Cu1—N1^v^	86.2 (4)
O6'—W1—O4	92.5 (4)	N1—Cu1—N1^v^	166.4 (5)
O6—W1—O4	81.2 (4)	N2—Cu1—O1W	86.1 (3)
O1—W1—O5'	108.3 (6)	N2^v^—Cu1—O1W	86.1 (3)
O3—W1—O5'	81.8 (5)	N1—Cu1—O1W	96.8 (3)
O6'—W1—O5'	140.6 (6)	N1^v^—Cu1—O1W	96.8 (3)
O6—W1—O5'	158.1 (6)	N2—Cu1—O4	93.9 (3)
O4—W1—O5'	90.2 (4)	N2^v^—Cu1—O4	93.9 (3)
O1—W1—O5	91.4 (5)	N1—Cu1—O4	83.2 (3)
O3—W1—O5	95.9 (4)	N1^v^—Cu1—O4	83.2 (3)
O6'—W1—O5	158.3 (6)	O1W—Cu1—O4	180.000 (2)
O6—W1—O5	162.3 (6)	W1—O3—W1^i^	162.3 (6)
O4—W1—O5	81.2 (4)	W1—O4—W1^v^	114.9 (4)
O5'—W1—O5	20.4 (4)	W1—O4—Cu1	122.5 (2)
O1—W1—O8^ii^	166.6 (4)	W1^v^—O4—Cu1	122.5 (2)
O3—W1—O8^ii^	87.3 (4)	O5'—O5—W1	79 (2)
O6'—W1—O8^ii^	59.2 (5)	O5'—O5—W2	54.6 (19)
O6—W1—O8^ii^	74.6 (5)	W1—O5—W2	120.9 (7)
O4—W1—O8^ii^	75.0 (4)	O5—O5'—W2	107 (2)
O5'—W1—O8^ii^	83.8 (5)	O5—O5'—W1	80 (2)
O5—W1—O8^ii^	99.1 (5)	W2—O5'—W1	141.3 (8)
O1—W1—O8^iii^	164.6 (4)	O6'—O6—W1	76.9 (19)
O3—W1—O8^iii^	86.8 (4)	O6'—O6—W2^v^	58.5 (16)
O6'—W1—O8^iii^	84.3 (5)	W1—O6—W2^v^	121.0 (7)
O6—W1—O8^iii^	99.5 (5)	O6—O6'—W2^v^	101.1 (19)
O4—W1—O8^iii^	74.6 (4)	O6—O6'—W1	81.0 (19)
O5'—W1—O8^iii^	58.7 (5)	W2^v^—O6'—W1	140.3 (8)
O5—W1—O8^iii^	74.0 (5)	O7'^iv^—O7—W2	59.1 (11)
O8^ii^—W1—O8^iii^	25.3 (6)	O7'^iv^—O7—W2^iv^	62.2 (11)
O2—W2—O5'	107.3 (6)	W2—O7—W2^iv^	114.8 (7)
O2—W2—O7'^iv^	114.1 (5)	O7^iii^—O7'—W2^iii^	94.5 (13)
O5'—W2—O7'^iv^	138.6 (7)	O7^iii^—O7'—W2	91.4 (13)
O2—W2—O6'^v^	111.0 (5)	W2^iii^—O7'—W2	149.6 (7)
O5'—W2—O6'^v^	95.7 (6)	O8^i^—O8—Al1	73.4 (4)
O7'^iv^—W2—O6'^v^	70.1 (6)	O8^i^—O8—W1^ii^	78.3 (10)
O2—W2—O7'	111.5 (5)	Al1—O8—W1^ii^	124.6 (6)
O5'—W2—O7'	74.1 (6)	O8^i^—O8—W1^iv^	76.4 (10)
O7'^iv^—W2—O7'	90.2 (7)	Al1—O8—W1^iv^	123.7 (6)
O6'^v^—W2—O7'	137.4 (6)	W1^ii^—O8—W1^iv^	93.3 (4)
O2—W2—O6^v^	93.7 (5)	O8^i^—O8—W2^iv^	171.0 (3)
O5'—W2—O6^v^	91.3 (6)	Al1—O8—W2^iv^	115.6 (5)
O7'^iv^—W2—O6^v^	86.5 (6)	W1^ii^—O8—W2^iv^	96.3 (4)
O6'^v^—W2—O6^v^	20.4 (5)	W1^iv^—O8—W2^iv^	96.9 (4)
O7'—W2—O6^v^	153.6 (6)	Al1—O9—W2^iii^	118.0 (5)
O2—W2—O5	91.5 (5)	Al1—O9—W2	118.0 (5)
O5'—W2—O5	18.6 (5)	W2^iii^—O9—W2	99.8 (6)
O7'^iv^—W2—O5	152.8 (6)	Al1—O9—W2^iv^	118.0 (5)
O6'^v^—W2—O5	92.8 (6)	W2^iii^—O9—W2^iv^	99.8 (6)
O7'—W2—O5	88.7 (6)	W2—O9—W2^iv^	99.8 (6)
O6^v^—W2—O5	82.5 (6)	Cu1—O1W—H1W	126.8
O2—W2—O7	89.1 (5)	N1—C1—C2	106.1 (9)
O5'—W2—O7	161.3 (7)	N1—C1—H1C	110.5
O6'^v^—W2—O7	86.3 (6)	C2—C1—H1C	110.5
O7'—W2—O7	91.8 (5)	N1—C1—H1D	110.5
O6^v^—W2—O7	96.7 (5)	C2—C1—H1D	110.5
O5—W2—O7	179.0 (6)	H1C—C1—H1D	108.7
O2—W2—O7^iii^	86.7 (5)	N2—C2—C1	108.6 (9)
O5'—W2—O7^iii^	89.9 (6)	N2—C2—H2C	110.0
O7'^iv^—W2—O7^iii^	92.2 (5)	C1—C2—H2C	110.0
O6'^v^—W2—O7^iii^	158.7 (6)	N2—C2—H2D	110.0
O6^v^—W2—O7^iii^	178.7 (5)	C1—C2—H2D	110.0
O5—W2—O7^iii^	98.7 (6)	H2C—C2—H2D	108.3
O7—W2—O7^iii^	82.1 (7)	C1—N1—Cu1	107.7 (7)
O2—W2—O9	153.2 (6)	C1—N1—H1A	110.2
O5'—W2—O9	89.3 (6)	Cu1—N1—H1A	110.2
O7'^iv^—W2—O9	52.5 (5)	C1—N1—H1B	110.2
O6'^v^—W2—O9	87.3 (6)	Cu1—N1—H1B	110.2
O7'—W2—O9	52.1 (5)	H1A—N1—H1B	108.5
O6^v^—W2—O9	107.2 (6)	C2—N2—Cu1	107.4 (7)
O5—W2—O9	107.5 (5)	C2—N2—H2A	110.2
O7—W2—O9	72.2 (5)	Cu1—N2—H2A	110.2
O7^iii^—W2—O9	72.1 (5)	C2—N2—H2B	110.2
O2—W2—O8^iii^	153.7 (4)	Cu1—N2—H2B	110.2
O5'—W2—O8^iii^	56.7 (6)	H2A—N2—H2B	108.5
O7'^iv^—W2—O8^iii^	84.6 (6)		

Symmetry codes: (i) −*x*, −*x*+*y*, −*z*+1/2; (ii) *y*−1, *x*+1, −*z*+1/2; (iii) −*x*+*y*−1, −*x*+1, *z*; (iv) −*y*+1, *x*−*y*+2, *z*; (v) *x*−*y*+1, −*y*+2, −*z*+1/2.

### Hydrogen-bond geometry (Å, °)

**Table:**

*D*—H···*A*	*D*—H	H···*A*	*D*···*A*	*D*—H···*A*
O1W—H1W···O2^vi^	0.85	2.25	2.856 (9)	128
N1—H1B···O5^vi^	0.90	2.26	3.138 (17)	163
N1—H1B···O5'^vi^	0.90	2.30	3.185 (17)	170
N2—H2A···O7^vii^	0.90	2.35	3.101 (17)	141
N2—H2B···O1^v^	0.90	2.11	2.956 (12)	157

Symmetry codes: (vi) *x*+1/3, *x*−*y*+5/3, *z*+1/6; (vii) −*x*+2/3, −*y*+7/3, −*z*+1/3; (v) *x*−*y*+1, −*y*+2, −*z*+1/2.

## References

[bb1] Brandenburg, K. (1999). *DIAMOND* Crystal Impact GbR, Bonn, Germany.

[bb2] Higashi, T. (1995). *ABSCOR* Rigaku Corporation, Tokyo, Japan.

[bb3] Hill, C. L. (1998). *Chem. Rev.* **98**, 1–2. 10.1021/cr960395y 11851497

[bb4] Lan, Y. Q., Li, S. L., Li, Y. G., Su, Z. M., Shao, K. Z. & Wang, X. L. (2008). *CrystEngComm*, **10**, 1129–1131.

[bb5] López, X., Maestre, J. M., Bo, C. & Poblet, J. M. (2001). *J. Am. Chem. Soc.* **123**, 9571–9576. 10.1021/ja010768z 11572677

[bb6] Lu, Y. K., Cui, X. B., Chen, Y., Xu, J. N., Zhang, Q. B., Liu, Y. B., Xu, J. Q. & &Wang, T. G. (2009). *J. Solid State Chem.* **182**, 2111–2117.

[bb7] Lu, Y. K., Cui, X. B., Liu, Y. B., Yang, Q. F., Shi, S. Y., Xu, J. Q. & Wang, T. G. (2009). *J. Solid State Chem.* **182**, 690–697.

[bb8] Lu, Y. K., Xu, J. N., Cui, X. B., Jin, J., Shi, S. Y. & Xu, J. Q. (2010). *Inorg. Chem. Commun.* **13**, 46–49.

[bb9] Lu, Y., Xu, J. & Yu, H. (2010). *Acta Cryst.* E**66**, m263–m264. 10.1107/S1600536810002473 PMC2983689 21580216

[bb10] Meng, F. X., Chen, Y. G., Pang, H. J., Shi, D. M. & Sun, Y. (2008). *J. Coord. Chem.* **61**, 1513–1524.

[bb11] Pope, M. T. & Müller, A. (1991). *Angew. Chem. Int. Ed. Engl.* **30**, 34–48.

[bb12] Rigaku (1998). *PROCESS-AUTO* Rigaku Corporation, Tokyo, Japan.

[bb13] Sheldrick, G. M. (2008). *Acta Cryst.* A**64**, 112–122. 10.1107/S0108767307043930 18156677

[bb14] Wang, J. P., Shen, Y. & Niu, J. Y. (2006). *J. Coord. Chem.* **59**, 1007–1014.

[bb15] Xu, Y., Xu, J. Q., Zhang, K. L., Zhang, Y. & You, X. Z. (2000). *Chem. Commun.* pp. 153–154.

[bb16] Yuan, M., Li, Y. G., Wang, E. B., Tian, C. G., Wang, L., Hu, C. W., Hu, N. H. & Jia, H. Q. (2003). *Inorg. Chem.* **42**, 3670–3676. 10.1021/ic026296m 12767207

[bb17] Yuan, L., Qin, C., Wang, X. L., Li, Y. G. & Wang, E. B. (2009). *Dalton Trans.* pp. 4169–4175. 10.1039/b818535b 19452066

